# Cross-Cultural Adaptation and Measurement Properties of the Upper Limb Functional Index (ULFI) for Greek-Speaking Patients

**DOI:** 10.7759/cureus.40029

**Published:** 2023-06-06

**Authors:** Grigorios Chamogeorgakis, Stefanos Karanasios, Georgios Theotokatos, Ioannis Vasilogeorgis, Vasileios Korakakis

**Affiliations:** 1 Physiotherapy, Hellenic OMT eDu, Athens, GRC; 2 Physiotherapy, University of West Attica, Athens, GRC; 3 School of Physical Education and Sport Science, National and Kapodistrian University of Athens, Athens, GRC; 4 Physiotherapy, King's College, London, GBR

**Keywords:** responsiveness, validity, reliability, upper-limb, outcome measure, ulfi

## Abstract

Introduction: The upper limb functional index (ULFI) is a widely used outcome measure for patients with upper limb musculoskeletal disorders (ULMSDs) that is available in several languages. Our purpose was to develop the Greek version of the ULFI and test its test-retest reliability, validity, and responsiveness in a cohort of patients with ULMSD.

Methods: We used a merged methodology of published guidelines and recommendations for the translation and cross-cultural adaptation process. One hundred patients with ULMSDs completed the ULFI-Gr on three occasions: baseline, 2-7 days later to evaluate repeatability, and 6 weeks later to assess responsiveness. Participants completed the quick disability of the arm, shoulder, and hand questionnaire (Quick-DASH) and a numerical pain rating scale (NPRS) to evaluate convergent validity. Also, a global rating of change (GROC) scale was used to evaluate responsiveness.

Results: Minor wording adaptations were required during the translation and cross-cultural adaption of the questionnaire. Factor analysis resulted in two main factors explaining 40.2% of the total variance. The ULFI-Gr was found to be reliable (intraclass correlation coefficient: 0.97, 95% confidence interval: 0.95-0.99) with a small measurement error (standard error of measurement: 3.34%, minimal detectable change: 7.79%). The ULFI-Gr showed a strong negative correlation with the Quick-DASH (-0.75), a moderate to strong negative correlation with the NPRS (-0.56), and a good level of responsiveness (standardized response mean: 1.31, effect size: 1.19).

Conclusions: The ULFI-Gr can be used as a reliable, valid, and responsive patient-reported outcome measure to evaluate the functional status of patients with ULMSDs.

## Introduction

Upper limb musculoskeletal disorders (ULMSDs) are a common cause of pain and disability leading to increased productivity loss and substantial healthcare burden [1]. ULMSDs may include various pathological conditions arising from the joints, tendons, ligaments, muscles, bones and neural tissue of the upper limb and occasionally the cervical or thoracic spine [2]. During the management of patients with ULMSDs, healthcare practitioners are required to provide a careful assessment of presented symptoms and activity limitations [3]. Therefore, using self-reported outcome measures is considered a practical and cost-effective way to provide an accurate prognosis, evaluate the outcome, and inform clinical decision-making [3].

Several region-specific upper-limb patient-reported outcome measures (PROMs) are available in the literature including the neck and upper limb index (NULI), the upper extremity functional index (UEFI), the upper extremity functional scale (UEFS), the disabilities of the arm, shoulder, and hand (DASH), the short version of DASH (Quick-DASH), and the upper limb functional index (ULFI) [3-4]. However, several concerns have been raised for these PROMs with regard to their measurement properties, for example, the validity of the Quick-DASH, the reliability of the UEFS, and the development methodology and content validity of the UEFS and the NULI [5-7]. Evidence suggests that one of the most easily administered and practical questionnaires providing good psychometric properties in patients with ULMSDs is the ULFI (Intraclass Correlation Coefficient [ICC]=0.98; standard error of measurement [SEM]=3.41, minimal detectable change [MDC90]=7.9) [8-10]. The ULFI consists of 25 items scored on a three-point Likert scale and has been designed to evaluate the patient’s functional status and level of participation in activities [11]. The ULFI has been translated and cross-culturally adapted in several languages such as Spanish, Turkish, French-Canadian, Italian, Korean, Brazilian Portuguese, Persian, and Urdu [2, 7, 9, 12-16].

The availability of PROMs in different languages is essential to improve everyday clinical practice and promote international research [17]. Based on published guidelines, translation and cross-cultural adaptation of PROMs requires certain steps to ensure comparability of language and similarity of interpretability between the original and the translated version, and subsequently the evaluation of the measurement properties of the new language version of the PROM [18-19]. To our knowledge, the ULFI has not been translated and cross-culturally adapted into Greek yet.

Therefore, the objectives of the present study were: (1) to translate and cross-culturally adapt the ULFI for Greek-speaking patients, and (2) to assess the measurement properties of the Greek version of the PROM in terms of reliability, validity, and responsiveness.

## Materials and methods

Prior to commencing the study, permission was granted from the PROM developers. Then, a merged methodology for translation and cross-cultural adaptation of PROMs was followed according to published recommendations [18-21].

Two bilingual translators (one with medical background and one “naive" to the questionnaire), whose native language was the target language (Greek), produced two independent ULFI translations. Subsequently, a research committee (the two translators and the investigators) synthesized the two forward translations into one using a consensus process.

Two different translators blinded to the concepts explored, whose native language was English and who were fluent in the target language, produced two independent back translations of the original version of the questionnaire. Then, the research committee reviewed the forward and back translations through a consensus procedure to develop the pre-final Greek version of the ULFI. During this process, the committee evaluated the comparability of language and similarity of interpretability [4].

The pre-final version of the Greek version of the ULFI was administered to a sample of 24 Greek-speaking individuals with ULMSD (12 men and 12 women) with an age range of 20-60 years old. After completing the questionnaire, participants were interviewed to assess the content validity of the PROM. The responders were interviewed by the principal researcher regarding the comprehensibility of each item; the clarity of the instructions and response options; and the relevance of the questionnaire to their musculoskeletal condition. Based on the results of the pre-testing procedure, the research committee produced the final version of the Greek version of the ULFI (ULFI-Gr).

Participants and procedures

Patients with ULMSDs were recruited from various physiotherapy clinics in Greece from June 2019 to June 2022. Patients were assessed for eligibility by a medical practitioner based on subjective and objective examinations. Participants were included if were older than 18 years old; have been diagnosed with an upper limb condition with symptoms duration of ≤12 weeks and were fluent in the Greek language. Exclusion criteria were: inability to read Greek; cancer; infectious, neurological disease, or other systemic diseases that could affect the upper limb. In addition, we recruited 20 healthy asymptomatic volunteers (>18 years old) for known group validity evaluation of the ULFI-Gr. All participants gave written informed consent.

At the initial visit, we recorded participants’ demographic characteristics including age, sex, height, weight, symptom duration, and painful side. To assess the convergent validity of the PROM, during the first session participants were asked to rate their worst experienced pain over the previous week using an 11-point numerical pain rating scale (NPRS) and subsequently, to complete the Greek versions of the ULFI and Quick-DASH questionnaire. To evaluate the test-retest reliability, ULFI-Gr was administered twice between 2 and 7 days after their first visit. All patients followed supervised physiotherapy sessions for six weeks (two sessions per week). Sessions were delivered by three musculoskeletal physiotherapists with more than 6 years of experience. To assess responsiveness, the PROM was administered for a third time (at 6 weeks) along with a six-point Likert-scale assessing global rating of improvement ranging from “much worse” to “completely recovered.” All questionnaires were completed in a quiet place without any assistance or feedback. Ethical clearance was approved by the University of Thessaly internal Ethics Committee (ID: 4-1/5-6-2019).

Measurement instruments

Upper Limb Functional Index (ULFI)

The ULFI includes 25 items that assess self-perceived activity limitations in patients with ULMSDs. Each item offers three response options, i.e. “Yes” (1 point), “Partly” (0.5 points), and “No” (0 points) [11]. The total points (from 0 to 25) are multiplied by four indicating the maximum disability. Then, this total score is subtracted from 100 to provide the patient’s functional score relative to their maximum or pre-injury function (0%: maximum limitation, 100%: normal or pre-injury function). No more than two missing responses are allowed for the calculation of the total score [11, 22]. The original English ULFI version has demonstrated excellent reliability (ICC=0.98; Cronbach’s α=0.92), high concurrent validity when compared to the Quick-DASH (r=0.86), and a minimal detectable change (MDC) of 7.9% in patients with ULMSD [22].

Quick-Disabilities of Arm, Shoulder, and Hand (Quick-DASH) Questionnaire

The Quick-DASH is a shorter version of the original 30-item DASH questionnaire. It contains 11 items that are scored using a five-point Likert scale ranging from 1 (no difficulty at all) to 5 (unable to do) and at least 10 out of the 11 items must be completed for the final score to be calculated [23]. The Greek version of the Quick-DASH has presented excellent internal consistency, test-retest reliability, and acceptable responsiveness [24].

Numerical Pain Rating Scale (NPRS)

Patients were asked to evaluate their worst pain the previous week from 0 (no pain) to 10 (worst pain ever). The NPRS has presented good reliability (ICC range: 0.74-0.76) with a minimally clinical important of difference (MCID) of 11% in patients with shoulder pain [25].

Global Rating of Change (GROC)

Participants were asked to evaluate the change of their condition regarding their upper limb symptoms using a Likert scale (-3: completely worse, -2: much worse, -1: little worse, 0: the same, 1: better, 2: much better, 3: completely recovered) at the 6 weeks following the intervention. The GROC has been extensively used in clinical research as a valid and reliable (ICC = 0.90) outcome measure [26].

Statistical analysis

Based on a sample size calculation (ICC>0.85; statistical significance p<0.05), a minimum sample of 91 participants was required for the study aims [13, 21]. To allow for a 10% loss to follow-up, and to ensure the stability of the variance-covariance matrix in the dimensionality analysis the sample size was finally set to 100 participants. The normal distribution of the data was checked using the Shapiro-Wilk test and Q-Q plots. We used descriptive statistics for the participants’ demographic characteristics and outcome measures. We used IBM SPSS Statistics (Version 25.0, IBM Corp., Armonk, NY) to analyze the data.

Validity

Seven bilingual physiotherapy researchers and 24 patients with ULMSD assessed the comparability of language and the similarity of interpretability. For the assessment, a Likert scale was used ranging from one (extremely comparable/similar) to seven (not at all comparable/similar). We used Aiken’s item-content validity coefficient (V) to analyze statistical significance (V coefficient > 0.70 corresponding to acceptable validity) [27].

To evaluate construct validity, we hypothesized that the asymptomatic and patient groups will score differently in the ULFI. We expected a statistically higher score for the healthy group compared to the patient group. We used a *t*-test to calculate differences between groups (patients with ULMSDs and healthy individuals).

The factorial validity of the ULFI-Gr was tested using an exploratory factor analysis (EFA) with varimax rotation. Eigenvalues of more than one and accounting for more than 10% of variance were extracted.

Pearson’s correlation coefficient (r) was used to evaluate convergent validity between the ULFI-Gr at baseline and the Greek versions of Quick-DASH and NPRS. Pearson’s correlation coefficient values ≥0.70, between 0.51 and 0.70, and ≤0.50 were considered as high, moderate and low, respectively [28]. We a priori hypothesized a strong correlation between the PROMs.

Reliability

Cronbach’s α was used for the evaluation of the internal consistency of the ULFI-Gr. Values of 0.70-0.95 were considered to indicate high internal consistency. ICC (two-way random model, absolute agreement) with a 95% confidence interval (CI) was used to evaluate test-retest reliability. We considered ICC values over 0.75 as excellent, between 0.4 and 0.75 as fair, and values less than 0.4 as poor (28). To assess absolute reliability, we calculated the SEM and MDC90. We recorded the time to complete the ULFI-Gr and evaluated the floor and ceiling effects of the PROM. Floor and ceiling effects were considered present if more than 15% of the participants scored the lowest (0) or the highest (100) possible score, respectively.

Responsiveness

Standardized response mean (SRM) and effect size (ES) were calculated for participants reporting improvement of their condition (GROC≥1) at the end of the 6-week physiotherapy management. SRM and ES values more than 0.80 were considered as large, between 0.51 and 0.80 as moderate and less than 0.50 as small. To evaluate the MCID we compared the results of the participants who reported an important change (‘much better’ or ‘completely recovered’) with those reporting a small change (‘better’) using the receiver operating characteristic (ROC) curve. Using the ROC curve, we evaluated the true-positive rate (sensitivity) compared to the false-positive rate (1-specificity). The area under the curve (AUC) illustrates the probability of discriminating between two classes (i.e., improved and not improved patients) ranging from 0.5 (not effective discrimination) to 1.0 (perfect discrimination). The MCID was determined as the optimal cut-off value of the ROC curve corresponding to the maximum of both sensitivity and specificity [29].

## Results

Translation, cross-cultural adaptation, and item content validity

Two linguistic discrepancies were identified during forward and backward translation and required cultural-linguistic adaptions. The expressions ‘irritable and/or bad tempered’ (items 14) and ‘dense objects’ (items 21) needed modifications to enhance comprehensiveness until a final consensus was reached by the translators and members of the expert committee. Twenty-four patients with ULMSDs were interviewed resulting in no issues regarding comprehensibility, comprehensiveness, and relevance of the items/responses of the ULFI-Gr.

Participants

A total of one hundred patients with ULMSDs (35 men and 65 women) with a mean age (±SD) of 46.7 (±14.9) years participated in the study. Participants’ demographic characteristics are presented in Table 1. The responders required 6-8 min to complete the ULFI-Gr. 

**Table 1:** Demographic and clinical characteristics of participants (N=101). ULFI-Gr, upper limb functional index - Greek; SD, standard deviation; Quick-DASH, Quick - disability of the arm, shoulder, and hand questionnaire; NPRS, numerical pain rating scale; N, sample

Characteristic	Mean ± SD (range) or No (percentage)
Age (years)	46.75 ± 14.9 (18-77)
Sex	
Men	35 (35%)
Women	65 (65%)
Diagnosis	
Rotator cuff related pain	39%
Lateral/medial epicondylitis	17%
Frozen shoulder	14%
Distal radial fracture	12%
De Quervain tenosynovitis	11%
Other	7%
Height (cm)	169 ± 9.2 (150-193)
Weight (kg)	69.7 ± 14.55 (47-110)
ULFI-Gr (%)	66.2 ± 18.4
Quick-DASH (%)	42.02 ± 18.8
NPRS (0-10)	5.5 ± 1.9

Validity

The known group validity analysis showed that patients with ULMSDs (mean score ± SD: 66.2 ± 18.4) scored significantly lower (p < 0.001) than the healthy individuals (mean score ±: 99.03 ± 2.4).

**Figure 1:**
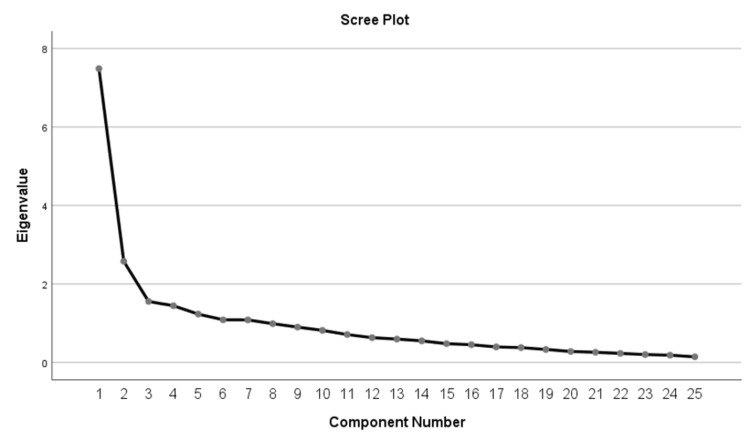
Scree plot from exploratory component analysis of the ULFI-Gr. ULFI-Gr, Greek version of the ULFI

The EFA of the ULFI-Gr resulted in a seven-factor solution with eigenvalues > 1 (Kaiser-Meyer-Olkin value = 0.796, p < 0.001; Bartlett’s sphericity test C2=1073.307, p < 0.001). Eigenvalues and the variance of each factor are presented in Table 2. Two factors explained 40.2% of the total variance (29.9% and 10.3%, respectively) while four items could not be added in any specific factor (Table 2, Figure 1).

**Table 2:** Factorial analysis of the ULFI-GR and loading of each item (factor loadings > 0.4 are in bold). ULFI-Gr, Greek version of the ULFI

	Factor 1	Factor 2	Factor 3	Factor 4	Factor 5	Factor 6	Factor 7
Eigen value	7.488	2.578	1.555	1.446	1.232	1.087	1.084
%Variance	29.953	10.311	6.22	5.782	4.927	4.346	4.337
Cumulative	29.953	40.265	46.484	52.266	57.194	61.540	65.877
Item 1	0.368	-0.312	0.101	-0.144	0.154	-0.433	0.545
Item 2	0.392	0.316	0.324	-0.355	0.390	-0.167	-0.290
Item 3	0.542	-0.061	-0.314	-0.334	0.089	0.108	-0.121
Item 4	0.676	-0.098	0.096	-0.374	-0.099	-0.209	0.035
Item 5	0.627	-0.214	-0.187	-0.132	0.154	0.166	-0.165
Item 6	0.389	0.458	0.285	-0.039	0.091	0.350	0.039
Item 7	0.553	-0.066	0.023	-0.146	-0.268	-0.114	-0.134
Item 8	0.410	-0.053	0.381	-0.252	0.035	0.552	0.141
Item 9	0.332	0.429	-0.140	-0.150	-0.307	0.023	0.535
Item 10	0.646	0.082	-0.382	-0.297	0.131	0.147	0.037
Item 11	0.542	0.454	0.232	-0.091	0.009	-0.151	-0.274
Item 12	0.440	0.231	-0.349	0.241	0.238	-0.180	0.013
Item 13	0.659	-0.059	-0.274	0.013	-0.129	-0.042	0.169
Item 14	0.573	0.273	0.122	-0.041	-0.269	-0.289	-0.202
Item 15	0.631	0.321	0.224	0.070	-0.353	-0.116	0.100
Item 16	0.660	0.042	0.371	-0.101	0.318	0.074	0.128
Item 17	0.448	0.421	-0.111	0.563	0.279	-0.004	-0.048
Item 18	0.479	0.547	0.044	0.037	-0.061	0.271	0.009
Item 19	0.258	0.676	-0.010	0.239	0.110	0.174	0.192
Item 20	0.598	0.494	0.142	0.258	0.291	-0.133	-0.027
Item 21	0.652	0.277	-0.108	0.333	-0.056	-0.031	-0.052
Item 22	0.481	-0.120	0.562	0.130	0.326	-0.075	0.225
Item 23	0.729	-0.058	-0.122	-0.008	-0.192	-0.032	-0.256
Item 24	0.598	-0.446	0.272	0.252	-0.192	0.113	-0.024
Item 25	0.647	0.016	-0.022	0.340	-0.300	0.154	-0.052

The ULFI-Gr showed a strong negative correlation with the Quick-DASH (r=-0.752; p < 0.001) (Table 3). A moderate to strong negative correlation was found between the ULFI-Gr and NPRS (r = -0.568, p < 0.001) (Table 3). No ceiling and floor effects were identified. 

**Table 3:** Test-rest reliability, internal consistency, and convergent validity of the ULFI-Gr. ULFI-Gr, upper limb functional index - Greek; ICC, intraclass correlation coefficient; SEM, standard error of measurement; MDC, minimal detectable change; CI, confidence interval; N, sample size; SRM, standardized response mean; ES, effect size

	Cronbach’s α N=100	ICC (95%CI) N=88	SEM% N=88	MDC_90_% N=88	Pearson correlation (Quick-DASH) N=100	Pearson correlation (NRPS) N=100	SRM	ES
ULFI-Gr	0.895	0.97 (0.95-0.99)	3.34	7.79	-0.752	-0.568	1.31	1.19

Reliability

Twelve participants were excluded from the test-retest reliability analysis due to significant changes in their symptoms between the administrations. The test-retest reliability of the ULFI-Gr was found excellent (ICC=0.97; 95% CI = 0.95-0.99). In terms of internal consistency, the questionnaire presented a high Cronbach's α (0.89). The SEM was 3.34 with an MDC90 of 7.79 (Table 3).

Responsiveness

**Figure 2:**
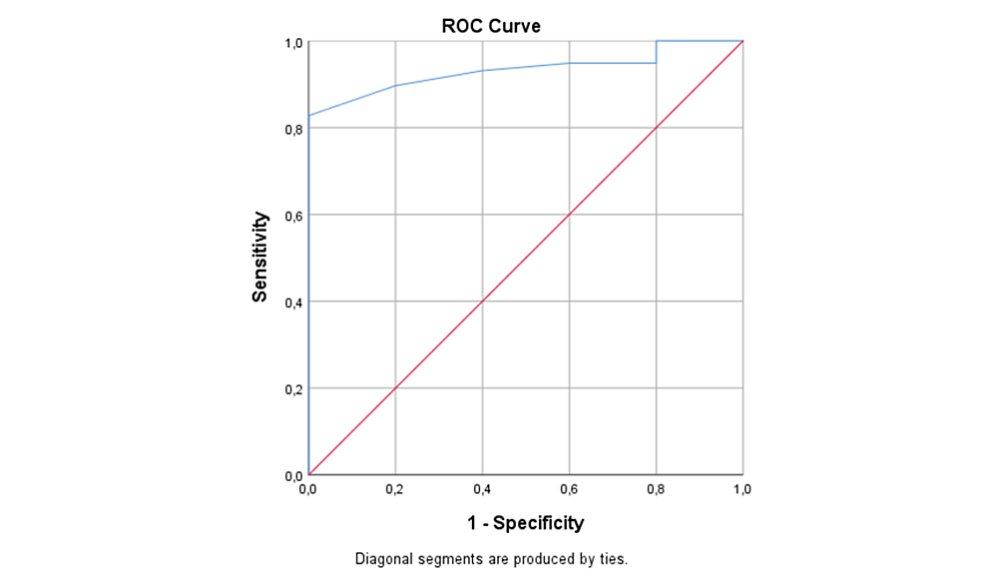
ROC curve of the ULFI-Gr. ROC, receiver operating characteristic; ULFI-Gr, Greek version of the ULFI

The ULFI-Gr presented adequate responsiveness with an SRM of 1.31 and an ES of 1.19 (Table 2). The AUC calculated to estimate the MCID for the ULFI-Gr was 0.933 (95%CI = 0.86-0.99) suggesting an excellent discriminative ability and the best cut-off point for the ULFI-Gr was 73 points (sensitivity = 87%; specificity = 80%) (Figure 2).

## Discussion

Our findings suggest that the ULFI has been successfully translated and cross-cultural adapted into the Greek language. The ULFI-Gr presented adequate face and content validity and excellent reliability in patients with ULMSDs. The factor analysis reflected the need for further exploration of the structure of the PROM. The correlations of the questionnaire compared to the Quick-DASH and NPRS were found ‘high’ and ‘moderate to high’, respectively. Notably, the ULFI-Gr was found highly responsive with a large effect size. The clinometric properties of the questionnaire were comparable to the other translated and cross-culturally adapted versions (Table 4). 

**Table 4:** Measurement properties of translated ULFI versions. ULFI, upper limb functional index; ICC, intraclass correlation coefficient; DASH, disability of the arm, shoulder, and hand; SEM, standard error of measurement; MDC, minimal detectable change; SRM, standardized response mean; EQ-5D, Euro Quality of life 5 dimensions; NPRS, numerical pain rating scale; SF-12, short form-12

Version	Reproducibility (ICC)	Internal consistency (Cronbach’s alpha)	Measurement error (%)	Responsiveness	Convergent validity	Ceiling and floor effects	Factor analysis
Arabic	-	0.88	-	-	-0.80 (DASH) -0.52 (NPRS)	0 and 0	-
Persian	0.92	0.91	SEM:3.11 MDC_90_:7.25	-	-0.71 (DASH)	0 and 0	One-factor structure (38% of variance)
French-Canadian	0.97	-	SEM:4 MDC_90_:9.3	ES: 0.62 SRM: 0.88	-0.64 (DASH)	-	-
Greek	0.97	0.89	SEM:3.34 MDC_90_:7.79	ES: 1.19 SRM: 1.31	-0.75 (Quick-DASH) -0.56 (NPRS)	0 and 0	Two-factor structure (29.3% and 10.3% of variance)
Italian	0.94	0.90	SEM:5 MDC_90_:12	-	-0.81 (DASH)	-	-
English	0.98	0.92	SEM:3.41 MDC_90_:7.9	ES: 0.93 SRM: 1.33	-0.82 (Quick-DASH)	0 and 0	One-factor structure (33.4% of variance)
Korean	0.90	0.94	-	-	-0.72 (DASH)	-	-
Spanish	0.93	0.94	SEM:3.52 MDC_90_:8.03	-	-0.59 (EQ-5D-3L)	-	One-factor structure (48% of variance)
Urdu	0.91	0.94	SEM: 3.89 MDC_95_: 10.6	-	-0.84 (Quick-DASH) -0.52 (NPRS) -0.69 to -0.76 (SF-12)	-	Two-factor structure (44.1% & 13.09% of variance)
Turkish	0.72	0.88	SEM: 2.94 MDC_95_: 5.35	-	-0.87 (DASH)	-	Two-factor structure (18.1% & 13.1% of variance)

Translation and cross-cultural adaptation of the questionnaire were derived from a rigorous approach using a well-established methodology from published recommendations [18-20]. We found two linguistic discrepancies between the English and the Greek version (items 14 and 21) which were literally and culturally adjusted according to the suggestions made by the expert committee. Although the structural analysis of ULFI-Gr indicated a seven-factor solution, two factors included 21 out of the 25 items explained, 40.2% of the total variance. The results presented herein were similar to the Urdu and Turkish versions that revealed also two dominant factors explaining 54% and 31.2% of the total variance, respectively with items that could not be added in any factor [16, 30]. Interestingly, the English and Spanish version structure evaluation suggested unidimensionality for the PROM [12, 22]. As such, a firm conclusion could not be argued regarding the underlying structure of the questionnaire and the data suggest that more rigorous statistical approaches are needed in the exploration of the PROM’s structure. Future studies using the Modern Test Theory approach which includes a collection of statistical models including confirmatory factor analysis, item response theory, and Rasch analysis should further evaluate the underlying structure of the PROM [31].

Despite the fact that an optimal value for Cronbach α coefficient remains unclear, evidence suggests that values between 0.70 and 0.95 are considered acceptable [19]. The ULFI-Gr presented a high internal consistency (Cronbach’s α: 0.89) which was similar to the English (0.92), Arabic (0.88), Persian (0.91), Italian (0.94), Spanish (0.93), and Turkish (0.88) version (Table 4) [2, 9, 12, 15-16, 22]. Similarly, the test-retest reliability of the ULFI-Gr was excellent (ICC=0.97) and similar to the French-Canadian (ICC=0.97) and English (ICC=0.98) versions (Table 4) [13, 22]. Other translated versions of the questionnaire presented lower ICC values which ranged between 0.82 and 0.94 (Table 4) and probably were influenced by the patient/sample configuration. These discrepancies may be attributed to several factors that may influence test-retest reliability analysis such as the time interval between administrations, patient condition, and the risk of recall bias. To illustrate, the Turkish version for example presented the lowest test-retest reliability (ICC=0.82) and plausibly this could be explained by the inclusion of patients with acute and subacute ULMSD symptoms which may have been significantly improved between the administrations [30]. In the present study, we included patients with chronic ULMSDs (≥12 weeks) and a time interval between 2 and 7 days was used between test-retest measurements to ensure condition stability. Nevertheless, 12 patients reported a significant change in their symptoms (GROC>1) between the administrations and therefore, they were excluded from test-retest reliability analysis.

Evidence suggests that there is a strong correlation between the ULFI and other upper-limb region-specific PROMs such as the DASH and Quick-DASH questionnaires [2, 7, 9, 12, 15-16, 22]. The correlation found between the ULFI-Gr and Quick-DASH questionnaire was high (-0.75) and consistent with the original version (-0.82) [22]. On the contrary, a moderate correlation between the ULFI-Gr and NRPS (-0.56) was found, an observation similar to the Urdu (0.52) and Spanish (0.52) translations and cross-cultural adaptations [12, 16] indicating that the PROM does not measure only the pain construct, but also disability. Also, similarly to all the other published versions, the ULFI-Gr presented no floor and ceiling effects.

For a PROM to be clinically useful, it must first be psycho-metrically sound but also must be able to detect the real change in health status (sensitivity to change) and display the ability to detect the absence of change when there is no real change (specificity to change) [31]. The ULFI-Gr was found able to detect large treatment effects (ES=1.19; SRM=1.31) following a 6-week physiotherapy intervention in patients with chronic ULMSDs. The effect sizes presented in our population were comparable to the ones presented for the original ULFI (ES=0.93; SRM=1.33) [22]. However, the responsiveness and the MCID of a PROM are context-specific, not fixed properties of a PROM, and are dependent on characteristics of the population, condition severity, chronicity, intervention, and period of follow-up [31]. For example, the responsiveness of the French-Canadian version displayed lower effect size values (ES=0.62; SRM=0.88) which could be explained by study population differences (acute, subacute, and chronic conditions) and the duration of the intervention (2 compared to 6 weeks) [13]. On top of that, using shorter time intervals between assessments for acute, subacute, and chronic patients may result in interpretation errors as acute patients show greater clinical changes than chronic patients in the same time frame [13]. The optimal cut-off point for the ULFI-Gr was found at 73% with sensitivity and specificity at 87% and 80%, respectively. Considering that a total score of 0 indicates the worst function and 100 is the maximum or pre-injury function, a large improvement was considered as a change of 26% or more in the total score of the PROM. Based on the authors’ knowledge, this is the first study presenting an MCID for the ULFI in patients with ULMSDs.

Limitations and future research

The present findings should be interpreted in light of some limitations. First, we decided to use a time interval between 2 and 7 days to ensure that the patient’s condition has not changed between test-retest administration. However, such a short time period between measurements may have substantially increased the risk of recall bias in reliability analysis [32]. Our sample consisted of patients with ULMSDs with symptom duration >12 weeks; therefore, the present findings cannot be generalized to acute or subacute conditions and we acknowledge that as another limitation. Further research is required to investigate if the psychometric properties of the ULFI-Gr differ in patients with acute ULMDs, as well as the underlying structure of the PROM.

## Conclusions

The Greek version of the ULFI has satisfactory content validity and is equivalent to the original version. It presents a high internal consistency, excellent test-retest reliability, and a strong negative correlation with the Quick-DASH questionnaire. The structural validity of the ULFI-Gr presents inconsistencies regarding factor structure when compared to the original version of the questionnaire. Nevertheless, the ULFI-Gr shows adequate responsiveness which is comparable to the English version. Based on our findings, the ULFI-Gr is a comprehensible, easy to use outcome measure with sound psychometric properties for Greek-speaking patients with ULMSDs.
